# *Haematospirillum jordaniae* Cellulitis and Bacteremia

**DOI:** 10.3201/eid2810.220326

**Published:** 2022-10

**Authors:** Emil Pal, Iztok Štrumbelj, Tjaša Cerar Kišek, Marko Kolenc, Mateja Pirš, Katarina Resman Rus, Tina Triglav, Tatjana Avšič-Županc

**Affiliations:** Murska Sobota General Hospital Department of Infectious Diseases, Murska Sobota, Slovenia (E. Pal);; National Laboratory of Health, Environment and Food, Murska Sobota (I. Štrumbelj);; Institute of Microbiology and Immunology, Ljubljana, Slovenia (T. Cerar Kišek, M. Kolenc, M. Pirš, K. Resman Rus, T. Triglav, T. Avšič-Županc)

**Keywords:** Haematospirillum jordaniae, cellulitis, bacteremia, bacteria, bacterial infections, skin infections, emerging pathogen, common reed

## Abstract

We isolated *Haematospirillum jordaniae* from a positive blood culture from a 57-year-old man in Slovenia who had bacteremia and bullous cellulitis of lower extremities. The infection was successfully treated with ciprofloxacin. Our findings signal the need for increased awareness about the clinical course of *H. jordaniae* and its potential effects as a human pathogen.

A 57-year-old man living near Lendava, Slovenia, with a medical history of type 2 diabetes, varicose veins in his legs, obesity, and arterial hypertension, sought treatment for a 1-day history of bilateral swelling, redness, warmness, and pain in his lower extremities. The day before, he had pricked himself on his left shin and the sole of his right foot with a reed in the Pacsa Fishing Lake in Hungary. At hospital admission, the patient was febrile (38.5°C) but with vital signs within reference ranges. 

Physical examination revealed painful, indurated, erythematous lower extremities, with edema and warmth. Clinically relevant results from blood analysis demonstrated leukocytosis (16.5 × 10^9^ cells/L) with neutrophilia (14.0 × 10^9^ cells/L) and elevated C-reactive protein (CRP; 189 mg/L), suggesting bacterial etiology; procalcitonin (PCT) level was within reference range (0.1 μg/L). We empirically introduced therapy with intravenous flucloxacillin (2 g/6 h) for coverage of cellulitis. 

On day 2 of hospitalization, extensive bullous changes appeared in the lower extremities. Because of unusual bilateral presentation, we added intravenous therapy with ciprofloxacin. Two days later, fever subsided, and blood leukocyte count returned to normal (10.5 × 10^9^ cells/L). CRP had mildly increased to 204 mg/L; PCT remained within reference range (0.4 μg/L). On day 7 of hospitalization, we observed major improvement in the patient’s laboratory parameters (leukocyte count 6.4 × 10^9^ cells/L, CRP 35 mg/L). We continued treatment with intravenous flucloxacillin and ciprofloxacin until discharge on day 13. Signs of bullous cellulitis in the lower extremities had subsided. 

Aerobic blood culture bottle was positive after 3 days of incubation. We observed small, slender, pleomorphic bacilli and coccobacilli in Gram stain. After subcultivation onto solid media, we detected growth on blood and chocolate agar (Figure, panels B, C) on the third day, with no growth observed on MacConkey or TCBS (Thiosulfate-citrate-bile salts-sucrose) agar or in microaerophilic atmosphere. However, we could not identify the causative agent using Gram stain from culture (Figure, panel A), colony morphology, growth characteristics, or MALDI-TOF (matrix-assisted laser desorption/ionization time-of-flight) mass spectrometry. We suspected *Francisella tularensis* on the basis of clinical manifestations and local epidemiology. We sent blood agar and chocolate agar plates to the reference Biosafety Level 3 laboratory at the Institute of Microbiology and Immunology (Ljubljana, Slovenia) for further analysis. We isolated DNA using QiaAmp DNA Mini Kit (QIAGEN, https://www.qiagen.com) and tested it, including dilutions from 1:10 to 1:1,000, by specific real-time PCR, which ruled out *F. tularensis* (*1*). We performed standard tube extraction protocol for MALDI-TOF mass spectrometry identification using the latest MALDI Biotyper sirius (Bruker Daltonics, https://www.bruker.com) and SR library according to manufacturer instructions but could not identify the organism because scores fell below genus cutoff values. We undertook further molecular analyses, included amplifying the 16S V3/V4 region using Mastermix 16S Complete (Molzym, https://www.molzym.com). We purified amplicons using QIAquick PCR purification kit (QIAGEN) and sequenced them on a ABI3500 genetic analyzer (Applied Biosystems, https://www.thermofisher.com). We analyzed 16S rDNA sequences using the CLC Main Workbench 21.0.5 (QIAGEN) and compared those sequences with others available in the rRNA databases: GenBank BLAST (https://blast.ncbi.nlm.nih.gov/Blast.cgi), Ribosomal Database Project (https://rdp.cme.msu.edu), and MicrobeNet (https://microbenet.cdc.gov). Our isolate most closely matched *Haematospirillum jordaniae* isolate Acr132, H5569 and H2509, with 100% sequence identity. By sequencing a longer, 1,462 bp 16S rRNA region (*2*), we observed 99.93% identity to *H. jordaniae* H2509 (GenBank accession no. OM075117). After successful molecular identification, we created *H*. *jordaniae* main spectra profiles according to manufacturer standard procedures and added them to a custom main spectra profile library because the pathogen was not part of any commercial mass spectra library (Appendix Figure). 

**Figure:**
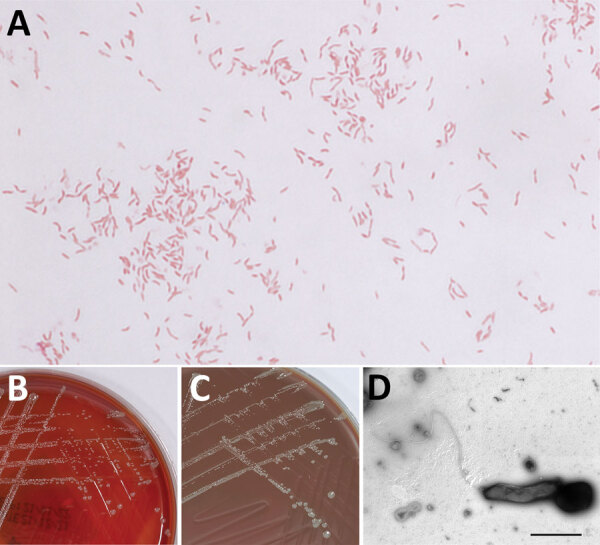
Detection of *Haematospirillum jordaniae* in a male patient in Slovenia. A) Gram stain of *H. jordaniae*; original magnification ×1,000. B) Colonies on blood agar after 3-day incubation. C) Colonies on chocolate agar after 3-day incubation. D) Transmission electron micrograph image of negatively stained cell of *H. jordaniae* exhibiting flagellum. Scale bar indicates 1 μm.

*H. jordaniae* is a slow-growing, gram-negative rod bacterium that is difficult to identify because it is not included in standard identification databases. Molecular analysis is necessary for definite identification (*3*,*4*). *H. jordaniae*, which belongs to the alphaproteobacteria family *Rhodospirillaceae* (*5*), was first identified as a potential human pathogen in 2016, when the new genus and species were described from an isolate obtained from a human blood sample in 2010 (*3*,*4*). An additional 13 isolates from human blood samples with identical or very similar 16S rRNA sequences, all from men (average age: 60), were later identified at the CDC Special Bacteriology Reference Laboratory (https://www.cdc.gov/ncezid/dhcpp/bacterial_special/special_lab.html).

We determined the antimicrobial susceptibility of *H. jordaniae* using gradient diffusion E-test strips (bioMérieux, https://www.biomerieux.com) and Liofilchem MTS (MIC test strips) for amoxicillin/clavulanic acid (https://www.liofilchem.com) on Muller-Hinton Fastidious agar (CO_2_, 48-h incubation). We interpreted results according to non–species-related EUCAST (https://www.eucast.org) PK/PD (pharmacokinetics/pharmacodynamics) antimicrobial susceptibility breakpoints (Table). According to the results of susceptibility testing, fluoroquinolones had the most favorable breakpoint-to-MIC ratios: ciprofloxacin and levofloxacin had MIC <0.002 mg/L (both) and PK/PD breakpoints of 0.25 mg/L (ciprofloxacin) and 0.5 mg/L (levofloxacin). 

**Table:** Antimicrobial susceptibility of *Haematospirillum jordaniae* from a male patient in Slovenia, interpreted according to non–species-related EUCAST PK/PD breakpoints*

Antimicrobial	MIC, mg/L	Susceptibility category
Benzylpenicillin	8	R
Ampicillin	2	S
Amoxicillin/clavulanic acid	0.125	S
Cefuroxime Iv	16	R
Cefotaxime	0.25	S
Imipenem	>32	R
Meropenem	1	S
Ciprofloxacin	<0.002	S
Levofloxacin	<0.002	S
Tigecycline	<0.016	S
*EUCAST, https://www.eucast.org. PK/PD, pharmacokinetics/pharmacodynamics; R, resistant; S, susceptible		

Molecular evidence of *H. jordaniae* in the blood of any vertebrate other than humans was described only in a bird species, the reed warbler, *Acrocephalus scirpaceus* (*6*). Possible routes of infection are through environmental contact, mostly following skin injury (*4*). Current knowledge about *H. jordaniae* is limited; therefore, our findings signal the need for increased awareness about its clinical course and potential effects as a human pathogen.

Appendix Additional information from study of *Haematospirillum jordaniae* cellulitis and bacteremia in a patient in Slovenia.
