# Supplementary material for: Haematospirillum jordaniae Cellulitis and Bacteremia
Source: Emerg Infect Dis. 2022 Oct;28(10):2116–9. doi: 10.3201/eid2810.220326 (PMC9514349; doi:10.3201/eid2810.220326)
Supplement: Appendix — Additional information from study of Haematospirillum jordaniae cellulitis and bacteremia in a patient in Slovenia. [file 22-0326-Techapp-s1.pdf]

Article DOI: https://doi.org/10.3201/eid2810.220326

Haematospirillum jordaniae Cellulitis and Bacteremia

Appendix

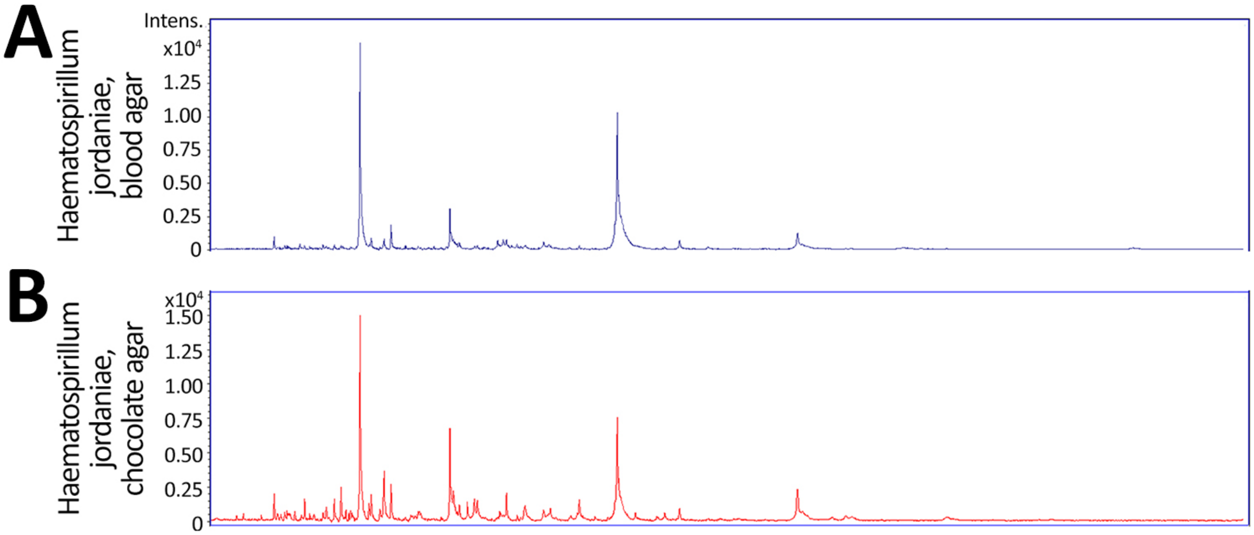

Appendix Figure. Mass spectra of *H. jordaniae* prepared from solid media after 3-day incubation
